# CircMAP3K11 Contributes to Proliferation, Apoptosis and Migration of Human Periodontal Ligament Stem Cells in Inflammatory Microenvironment by Regulating TLR4 via miR-511 Sponging

**DOI:** 10.3389/fphar.2021.633353

**Published:** 2021-02-18

**Authors:** Bohan Yu, Jiahui Hu, Qin Li, Fang Wang

**Affiliations:** Department of Periodontics, School and Hospital of Stomatology, Tongji University, Shanghai Engineering Research Center of Tooth Restoration and Regeneration, Shanghai, China

**Keywords:** human periodontal ligament stem cell, circMAP3K11, MiR-511-3p, TLR4, periodontitis

## Abstract

Growing number of studies regarding the role of circRNAs in the development of various diseases have emerged in recent years, but the role of circRNAs in periodontitis pathogenesis remains obscure. Human periodontal ligament stem cells (hPDLSCs) play a critical role in periodontal remodeling, regeneration and repair processes, and their regenerative capacity could be prohibited in local periodontal inflammatory microenvironment. Herein, we sought to uncover the molecular mechanisms of periodontitis pathogenesis by investigating the role of circMAP3K11 (hsa_circ_002284) for regenerative capacity of hPDLSCs under an inflammatory condition. The hPDLSCs isolated from periodontitis patients were used as a cell model of inflammatory microenvironment to study the effect of the circMAP3K11/miR-511-3p/TLR4 axis on the proliferation, apoptosis and migration of hPDLSCs under inflammatory conditions. Compared to the periodontal tissues from normal subjects, those from periodontitis patients exhibited higher expression levels of circMAP3K11 and TLR4, and lower expression level of miR-511-3p. Both the expressions of circMAP3K11 and TLR4 were negatively correlated with the expressions of miR-511-3p in periodontitis. *In vitro* studies demonstrated that circMAP3K11 is capable of enhancing hPDLSCs proliferation and migration, and reducing the apoptosis of hPDLSCs. We also found that circMAP3K11 could up-regulate the expression of transcription factors that are closely related to periodontal regeneration (Runx2, OSX, ATF4, and BSP). RT-PCR and western blot showed that the inhibitory role of miR-511-3p on TLR4 expression could be reversed by circMAP3K11, which was in line with the results of bioinformatics tools and luciferase reporter assay. Meanwhile, both *in vitro* and *in vivo* studies indicated that circMAP3K11 could reverse the effects of miR-511-3p in periodontitis, which further confirmed that circMAP3K11 functioned as a ‘sponge’ of miR-511-3p to positively regulate the expression of TLR4. Taken together, our study preliminarily uncovered a circMAP3K11/miR-511-3p/TLR4 axis that regulates the function of hPDLSCs in periodontitis, providing novel insight and scientific base in the treatment of periodontal tissue regeneration based on stem cells.

## Introduction

As a kind of chronic inflammatory and destructive disorder, periodontal disease is the primary cause of tooth loss in adults (Kulkarni et al., 2014). Initially, the dental plaque of bacteria on the teeth and gingiva causes localized inflammation of the gingiva named as gingivitis. Without timely treatment, gingivitis progresses to periodontitis, which means that inflammation can penetrate deep into tissues and lead to the loss of supporting connective tissue and alveolar bone (Kinane et al., 2017). The formation of periodontal pocket is a hallmark of periodontitis, and the deep periodontal pocket can eventually lead to tooth loosening and loss (Al-Ghutaimel et al., 2014; AlJehani, 2014). It is considered that periodontitis is an inflammatory reaction induced by external infection, which invades and destroys periodontal tissue irreversibly, and causes them to lose the ability of periodontal regeneration, and therefore, the repair of bone defect is the key to periodontal regeneration (Hynes et al., 2012; Han et al., 2014). In the last decades, human dental mesenchymal stem cells have been proved to hold great promise for bone regeneration due to their capacity of osteogenic differentiation. The adult stem cells have characteristics of self-renewal, clonogenicity, and multipotency, and play crucial roles in maintaining tissue homeostasis (Pekovic and Hutchison, 2008). Upon the cell loss or tissue injury, the adult stem cells can be triggered to proliferate and differentiate into the required type of cells. However, the ability of endogenous stem cells for tissue regeneration can be prohibited in the inflammatory microenvironment (Mourkioti and Rosenthal, 2005; Koning et al., 2013). Multiple studies demonstrated that persistent inflammation could not only reduce the proliferation and migration of stem cells *in situ*, but also inhibit the potential of tissue regeneration and the repair of stem cells (Kizil et al., 2015; VanHook, 2019). Thus, the decline or even the lack of repair and regeneration ability of endogenous stem cells is an important factor leading to organic lesions of periodontitis.

In 2004, Seo et al. successfully isolated and identified periodontal ligament stem cells (PDLSCs) (Seo et al., 2004). Several previous studies revealed that PDLSCs can be induced *in vitro* to differentiate into several tissues such as periodontal ligaments, alveolar bone, blood vessels, and peripheral nerves (Huang et al., 2009), which are considered to play an important role in periodontal remodeling, regeneration and repair (Liu et al., 2008; Park et al., 2011b). In a previous study, it was indicated that inflammation obviously inhibits the osteoblastic differentiation of PDLSCs (Park et al., 2011a). Another study also demonstrated that the self-renewal and multi-differentiation capacity of PDLSCs could be prohibited in the inflammatory microenvironment (Liu et al., 2014). Nevertheless, the molecular mechanism underlying the change in the function of PDLSCs in periodontitis remains obscure.

Toll-like receptor (TLR) plays key roles as pattern-recognition receptors in the recognition of specific molecular patterns that are present in microbial components. TLR4 is the best studied immune sensor that is expressed in all cell lines and was reported to function in promoting inflammatory response and the maturation and differentiation of immune cells, and regulating immune response (Potnis et al., 2013; Guijarro-Muñoz et al., 2014). It is reported that TLR4 is capable of regulating proliferation and osteogenic differentiation of bone marrow mesenchymal stem cells via Wnt3a/5a signaling (He et al., 2016). Gram-negative bacteria are the major pathogen involved in periodontitis, and their main pathogenic component is the lipopolysaccharide (LPS) that is primarily recognized by TLR4 (Janssens and Beyaert, 2003; Sun et al., 2008). A large body of evidence has proven the involvement of TLR4 in periodontitis (Renn et al., 2018; Liu et al., 2019a). Researchers have revealed that TLR4 can be activated by LPS to regulate NF-κB pathway of PDLSCs to subsequently decrease their osteogenic potential (Li et al., 2014). Recently, it was reported that the upregulation of TLR4 inhibits the osteogenic differentiation of PDLSCs in an inflammatory environment, suggesting the important role of TLR4 for regenerative capacity of PDLSCs in periodontitis (Liu et al., 2020). However, the specific regulatory mechanism needs to be further studied.

MicroRNAs (miRNAs), a kind of small non-coding RNAs, with about 19-to-24- nucleotides in length, can recognize specific target genes and regulate their expression, which involves them in various biological processes. Previous studies have found that several miRNAs play a regulatory role in osteogenesis and repair of bone defects (Ge et al., 2018; Guo et al., 2019b). Meanwhile, increasing studies reported that miRNAs play a key role in periodontal inflammation (Kebschull and Papapanou, 2015; Luan et al., 2017). Studies have demonstrated that miR-218 can attenuate osteoclast differentiation and inflammation response in periodontitis rats (Guo et al., 2019a) and the function of miR-21 in the regulation of osteogenic differentiation of PDLSCs was also conveyed (Wei et al., 2017). The expression of miR-511 in periodontitis gingiva was reported to be significantly higher than that in healthy gingiva (more than four fold) (Lee et al., 2011) and plays crucial roles in regulating TLR4 expression in several inflammatory diseases (Yang et al., 2013; Heinsbroek et al., 2016). Nevertheless, there are no reports on the role of miR-511/TLR4 in periodontitis. Circular RNAs (circRNAs) can act as natural miRNA sponge transcripts that compete for binding with endogenous RNAs in diverse species. The essential roles of circRNAs have been revealed in many diseases including cancer (Zhang et al., 2018), diabetes (Abbaszadeh-Goudarzi et al., 2020), and periodontitis (Zheng et al., 2021). The potential importance of interactions between circRNA and miRNA in disease regulation is known, but the roles of circRNA/miRNA interactions in periodontitis remain a largely unexplored field. To uncover the molecular mechanism involved in the functional phenotypic change of PDLSCs in periodontitis, it is crucial to integrate the circRNA-miRNA-mRNA competitive regulatory networks for understanding the effect of their interactions on the regenerative capacity of PDLSCs in periodontitis.

In this study, circMAP3K11 (hsa_circ_002284) was predicted to be an upstream regulator of miR-511 by using bioinformatics tools; we also found that the expression of circMAP3K11 was significantly different between healthy subjects and periodontitis patients. Based on this, we sought to explore the roles of circMAP3K11/miR-511/TLR4 axis for regenerative capacity of PDLSCs under inflammatory conditions to uncover the molecular mechanisms governing periodontitis pathogenesis. Our data revealed that circMAP3K11 prohibits the functions of PDLSCs via sponging miR-511-3p to enhance TLR4 expression in periodontitis environment, which provides novel insights and scientific information for clinical enhancement of periodontal tissue regeneration.

## Materials and Methods

### Clinical Specimen Collection

The study was approved by the Institutional Review Board of Affiliated Stomatology Hospital of Tongji University (Shanghai, China). All procedures involving human participants were conducted in accordance with the ethical standards of the Institutional Review Boards of Affiliated Stomatology Hospital of Tongji University, and with the 1964 Helsinki declaration and its later amendments or comparable ethical standards. Written informed consent was obtained from all individual participants included in the study. The normal periodontal ligament samples were collected from ten healthy patients who underwent impacted third molar extraction and/or orthodontic treatment, and the inflammatory periodontal ligament samples were collected from 20 periodontitis patients who had experienced tooth extraction. The exclusion criteria of patients were as follows: patients with a clear family history of genetic disease, history of chronic infectious diseases, systemic diseases, history of taking special drugs or smoking, dental and periapical diseases, and no acute infection. Diagnostic criteria of periodontitis were as follows: 1) redness and swelling of the gingiva on the surface of periodontal pocket or bleeding after probing; 2) probe depth > 3 mm and attachment loss > 1 mm; 3) X-ray showed horizontal or vertical resorption of alveolar bone.

### Immunohistochemistry Assay

After fixation of periodontal ligament samples in 4% paraformaldehyde (PFA) for one day at 4°C, the samples were embedded in paraffin and cut into 4 μm thick slices. The blockage of slices was carried out for 30 min in 0.5% BSA. Next, the slices were incubated with anti-TLR4 antibody for 12 h, followed by the secondary antibody for 35 min. Then, the Dako REAL™EnVision™ Detection System was employed to visualize the immune complexes. Finally, the slides were observed and imaged under the Leica DM5000 microscope after counterstaining of nuclei by hematoxylin.

### Human Periodontal Ligament Stem Cell (hPDLSC) Isolation and Cultivation

The isolation and characterization of hPDLSCs were performed according to our previous study (Yu and Wang, 2014). The hPDLSCs were maintained in Dulbecco’s modified Eagle Medium (DMEM) mixed with 10% fetal bovine serum (FBS). The passage three of hPDLSCs was used to perform subsequent experiments.

### CircRNA Analysis and Target Prediction

TargetScan (http://www.targetscan.org/vert_72/) (Agarwal et al., 2015) was employed to determine the interaction sites between miR-511-3p and TLR4. The miRanda domain (Enright et al., 2003) (http://www.microrna.org/microrna/home.do) was used to predict the circMAP3K11/miRNA interaction.

### Cell Transfection

The small interfering RNA targeting circMAP3K11 (si-circMAP3K11), circMAP3K11 overexpression vector, miR-511 mimic, miR-511 inhibitor, TLR4 overexpression vector, and their controls were all purchased from RiboBio (Guangzhou, China). According to the manufacturers’ protocol, the transfection was performed in hPDLSCs by using the Lipofectamine^®^ 2000 Transfection Reagent kit. The transfection was conducted at 37°C for 48 h.

### Cell Proliferation Assay

The cell proliferation was detected by 3-(4, 5-dimethylthiazol-2-yl)-2, 5-diphenyltetrazolium bromide (MTT) reagent. In brief, the transfected PDLSCs were seeded in a 96-well plate at a density of 8×10^3^ cells/well and cultured in an incubator containing 5% CO_2_ at 37°C. After certain times of incubation (0, 24, 48, and 72 h), the MTT reagent at a concentration of 0.2 g/L was added into each well followed by incubation for further 4 h. Finally, the viability of cells in each well was evaluated by the optical density measurement at 570 nm with a microplate reader.

### Colony Formation Assay

The hPDLSCs (2.5×10^2^) were seeded into 60-mm dish and cultured in a 5% CO_2_ incubator at 37°C. The cultured media were replaced with fresh media twice a week. After incubation for 14 days, the cells were washed twice with cold PBS and fixed in 4% PFA, followed by staining with 2.5% crystal violet. The dishes were rinsed thrice using tap water to remove the dye before counting stained colonies.

### Transwell Assay

The migration ability of hPDLSCs was evaluated by Transwell chamber (Millipore, Billerica, MA, UNITED STATES). After resuspending hPDLSCs with DMEM at a density of 8×10^4^ cells/mL, the cell suspension was placed in the upper chamber and DMEM with 10% FBS was added into the lower chamber. Then, the Transwell chamber was placed in a 5% CO_2_ incubator at 37°C for 24 h. The cells that did not migrate were removed from the membrane, the remaining cells in membrane were fixed with 4% PFA for 10 min and stained with 0.5% crystal violet. The stained cells were gently rinsed with tap water before counting under an optical inverted microscope.

### Annexin V-FITC/PI Double Staining Assay

Apoptosis of hPDLSCs was detected by flow cytometry after staining with Annexin V-FITC/PI. Briefly, 2×10^4^ cells were seeded in the six well plate per well and incubated in a 5% CO_2_ incubator at 37°C. 24 h later, the cells were harvested and rinsed with cold PBS, followed by double-staining with Annexin V-FITC and PI for 20 min in the dark. Finally, flow cytometry was employed for quantification of apoptotic cells.

### Quantitative Real-Time PCR Analysis

By using the TRIzol reagent, total RNA was extracted from PDLSCs. The Reverse Transcription kit was applied to reverse transcribe RNA into cDNA. Afterward, the specific cDNA was subsequently used to perform qPCR reaction. The procedure of qPCR reaction was as follows: initial-denaturation for 5 min at 95°C, followed by 40 cycles, namely, denaturation for 10 s at 95°C, annealing for 30 s at 60°C and extension for 20 s at 75°C. In this study, β-actin was used as the internal reference for circMAP3K11 and TLR4 whereas U6 was used as internal reference for miR-511. The relative expression of circMAP3K11, miR-511 and TLR4 was calculated based on the 2^−ΔΔCT^ (Livak and Schmittgen, 2001). The primer sequences for qRT-PCR were listed in Table 1.

**TABLE 1:** Primer sequences for RT-PCR.

Gene	Forward primer (5′-3′)	Reverse primer (5′-3′)
circMAP3K11	AAG​CTG​TCT​CCC​CTG​GAG​C	CGG​GAC​CTT​CTC​CTC​CCA​TT
miR-511	AGT​GCT​GGT​GTC​TTT​TGC​TCT​G	TAT​GGT​TGT​TCA​CGA​GTC​CTT​CAC
TLR4	GGT​GCC​TCC​ATT​TCA​GCT​CT	GAT​GAA​GTG​CTG​GGA​CAC​CA
Runx2	ATG​CTT​CAT​TCG​CCT​CAC​AAA	GCA​CTC​ACT​GAC​TCG​GTT​GG
OSX	GCC​AGA​AGC​TGT​GAA​ACC​TC	GCT​GCA​AGC​TCT​CCA​TAA​CC
ATF4	AGT​CGG​GTT​TGG​GGG​CTG​AAG	TGG​GGA​AAG​GGG​AAG​AGG​TTG​TAA
BSP	GCG​AAG​CAG​AAG​TGG​ATG​AAA	TGC​CTC​TGT​GCT​GTT​GGT​ACT​G
β-actin	TCC​CTG​AGA​CGC​TAG​ATG​AGG	CGT​TTA​GCA​GTT​TTG​TCA​GCT​C
U6	CTCGCTTCGGCAGCACA	AAC​GCT​TCA​CGA​ATT​TGC​GT

### Western Blot Analysis

The PDLSCs were lyzed with RIPA on ice and the protein concentration was determined by a BCA kit. Next, equal amounts of protein were purified by SDS-PAGE, followed by transfer onto the PVDF membrane. The membrane was blocked with skimmed milk for 1 h at 25°C before incubation overnight at 4°C with the specific primary antibodies. After that, the membrane was rinsed twice with TBST and incubated with the secondary antibody to bond the primary antibody at 25°C for 1 h. Finally, the band of specific protein was visualized by using ECL reagent and quantified by the ImageJ software. The protein β-actin was used as the internal reference in the present study.

### Dual-Luciferase Reporter Assay

The dual-luciferase reporter assay was performed to validate the targeted relation between miR-511 and circMAP3K11 (or TLR4). Following the manufacturer’s manuals, the miR-511 mimic and its negative control (mimic NC) were sub-cloned into the *Renilla* luciferase reporter vector psiCHECK-2. Then, circMAP3K11 (or TLR4) wild-type (WT) and mutant (MUT) dual-luciferase reporter vectors were constructed and used for co-transfection with each of the above *Renilla* luciferase reporter vector into HKE293 cells. The transfected cells were harvested after incubation for 48 h. By using a commercial Dual-Luciferase^®^ Reporter 1,000 Assay system, luciferase activity of the collected cells was detected. The relative luciferase activity was calculated based on the *Renilla* fluorescence activity as reference control.

### Establishment of the Periodontitis Model in Mice

All experiments involving animal in this study were approved by the Ethics Committee of the affiliated Stomatology Hospital of Tongji University. All animal studies were performed in accordance with the approval protocol of the Animal Care and Use Committee of the affiliated Stomatology Hospital of Tongji University. 24 female C57BL/6 J mice (8 weeks old, specific-pathogen-free grade) were used to establish the periodontitis mouse model as previously described (Yoshihara-Hirata et al., 2018). In short, a 6–0 non-absorbable silk thread infiltrated with *P. gingivalis* bacteria was used to ligate around the cervical portion of the maxillary second M, and the silk thread was replaced every two days. After successful establishment of periodontitis model, the mice were divided into four groups as follows: 1) control (periodontitis mice without any treatment); 2) si-circMAP3K11 (periodontitis mice with oral injection of the cells transfected with stable expression of si-circMAP3K11) (1 × 10^4^cells/mice); 3) miR-511-3p *antagomir* (periodontitis mice with oral injection of miR-511-3p *antagomir*) (200nM/mice); 4) si-circMAP3K11 + miR-511-3p *antagomir* (periodontitis mice treated with oral injection of si-miR-511-3p after injection of the si-circMAP3K11 transfected cells). After treatment for 48h, mice were sacrificed for collection of periodontal tissue samples. In this experiment, miR-511-3p *antagomir* was purchased from Genesky Biotechnologies (China).

### TUNEL and Ki-67 Assays

The TUNEL assay and Ki-67 assay were performed respectively to observe the cell apoptosis and proliferation of periodontal tissue in periodontitis mice with different treatment. The mice periodontal tissue from the four groups were fixed in 4% PFA and embedded in paraffin to cut into 3 μm-thick sections. Then, the sections were deparaffinized and hydrated, followed by permeabilization with 0.2% Triton X-100 at 4°C for 5 min. The apoptotic cells of tissues were stained by TUNEL reaction mixture for 1 h at 37°C in the dark. The proliferating cells of periodontal tissues were immunologically stained with the primary antibody against Ki67 and the FITC-labeled secondary antibody. Afterward, the DAPI dye (5 mg/ml) was utilized to counterstain cell nuclei of all sections for 3 min at 25°C. The stained sections were washed thrice, observed subsequently using fluorescence microscope.

### Statistical Analysis

All experiments in this study were performed at least three times, and all data were expressed as average ±standard deviation (SD). The unpaired Student t-test was used to examine the difference between the two groups, and one-way ANOVA with post-hoc test (Bonferroni) was used to analyze the difference among three groups. All statistical analysis was conducted on version 8.0.1 of GraphPad Prism (Graphpad software, Inc., La Jolla, UNITED STATES). *p* < 0.05 showed that the discrepancy was statistically significant.

## Results

### circMAP3K11 Is Highly Expressed in Periodontitis and Plays Roles in the Proliferation, Apoptosis and Migration of hPDLSC

**FIGURE 1:**
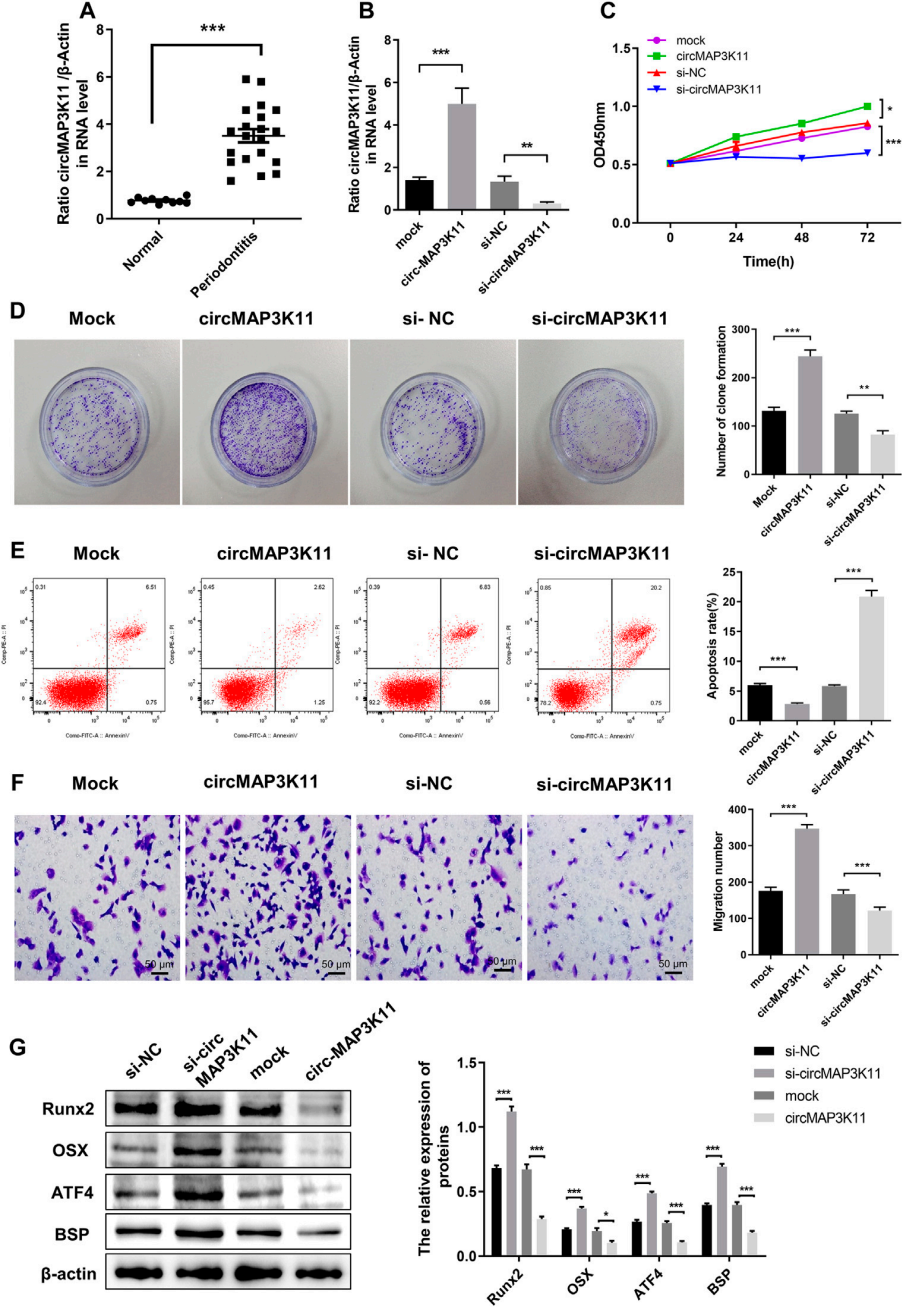
circMAP3K11 is overexpressed in periodontitis tissue and exert suppressing effect on regenerative capacity of PDLSCs **(A)** The expression level of circMAP3K11 in periodontitis and normal samples by RT-PCR **(B)** The expression level of circMAP3K11 in PDLSCs transfected with circMAP3K11 overexpression plasmid, siRNA targeted circMAP3K11, and corresponding control (mock and si-NC) **(C)** The cell viability of PDLSCs transfected with circMAP3K11 overexpression plasmid, siRNA targeted circMAP3K11, and corresponding control was determined by the MTT assay **(D)** The proliferation of PDLSCs transfected with circMAP3K11 overexpression plasmid, siRNA targeted circMAP3K11, and corresponding control was investigated by colony formation **(E)** The FCM was performed to detect the cell apoptosis of PDLSCs transfected with circMAP3K11 overexpression plasmid, siRNA targeted circMAP3K11, and corresponding control **(F)** Cell migration ability of PDLSCs transfected with transfected with circMAP3K11 overexpression plasmid, siRNA targeted circMAP3K11, and corresponding control was evaluated by transwell assay **(G)** Western blot showed the protein expression levels of Runx2, OSX, ATF4, and BSP in PDLSCs transfected with transfected with circMAP3K11 overexpression plasmid, siRNA targeted circMAP3K11, and corresponding control. Note: *p < 0.05, **p < 0.01, and ***p < 0.005.

As shown in Figure 1A, the expression of circMAP3K11 in periodontal ligament tissue was significantly different between periodontitis and non-periodontitis (normal) patients. To investigate the biological functions of circMAP3K11 in hPDLSC, hPDLSCs were transfected with circMAP3K11 expression plasmid, si-circMAP3K11, and their corresponding controls, respectively. The silencing and overexpression of circMAP3K11 was confirmed by RT-PCR. As shown in Figure 1B, the expression of circMAP3K11 in hPDLSCs was significantly increased after transfection of circMAP3K11 plasmid, but obviously reduced after transfection with si-circMAP3K11. MTT and colony formation assays showed that the viability and colony formation of hPDLSC were significantly promoted by overexpression of circMAP3K11 but remarkably suppressed by silencing of circMAP3K11 (Figures 1C,D). Meanwhile, flow cytometry was conducted to detect cell apoptosis after transfection of circMAP3K11 plasmid and si-circMAP3K11 (Figure 1E); and the result showed that overexpression of circMAP3K11 led to decreased cell apoptosis, while depletion of circMAP3K11 increased cell apoptosis in hPDLSC. Then, transwell assay showed that the cell migration ability of hPDLSC was also observably changed after transfection compared with controls, suggesting that circMAP3K11 could promote the hPDLSC migration (Figure 1F).

To preliminarily explore the effects of circMAP3K11 in osteogenic potential of hPDLSCs, the protein expression level of the Runx2, OSX, ATF4 and BSP was detected by western blot. As shown in Figure 1G, overexpression of circMAP3K11 could obviously increase the protein expression of Runx2, OSX, ATF4 and BSP in hPDLSCs while silencing of circMAP3K11 could significantly decrease Runx2, OSX, ATF4 and BSP protein levels in hPDLSCs.

These results demonstrated that circMAP3K11 may promote periodontitis by enhancing the viability, proliferation and migration of hPDLSCs, and reducing cell apoptosis of hPDLSCs. Our findings also suggested that circMAP3K11 is capable of promoting the osteogenic potential of hPDLSCs.

### miR-511-3p Directly Targeted circMAP3K11

**FIGURE 2:**
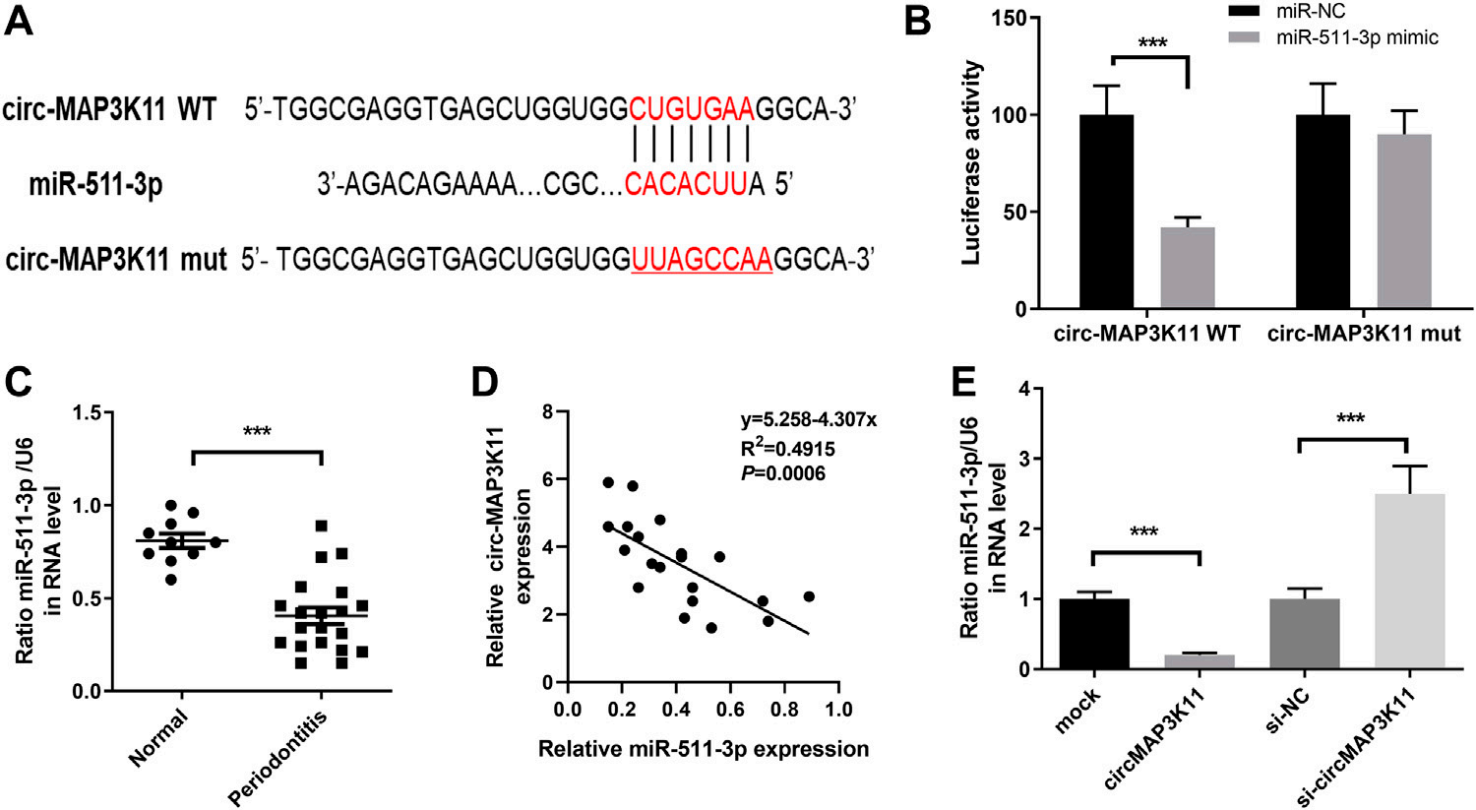
circMAP3K11 is a direct target of miR-511-3p **(A)** The putative complementary binding sites within circMAP3K11 and miR-511-3p **(B)** The dual-luciferase reporter assay was performed to validate that circMAP3K11 is a direct target of miR-511-3p **(C)** The expression level of miR-511-3p in periodontitis and normal samples was detected by RT-PCR **(D)** Spearman’s correlation analysis showed a negative correlation between circMAP3K11 and miR-511-3p expression in periodontitis samples **(E)** The expression of miR-511-3p in PDLSCs transfected with circMAP3K11 overexpression plasmid, siRNA targeted circMAP3K11, and corresponding control was detected by RT-PCR. Note: ***p < 0.005.

We found that circMAP3K11 can act as a sponge of miR-511 in the preliminary experiment of this study. In order to investigate the interaction between miR-511-3p and circMAP3K11, we predicted the putative binding sites of miR-511-3p in circMAP3K11 by bioinformatics tools (Figure 2A). The dual-luciferase reporter assay confirmed that miR-511-3p directly targets circMAP3K11 (Figure 2B). Next, we found that the expression level of miR-511-3p in periodontitis samples was significantly lower than those in normal sample (Figure 2C). The correlation analysis of circMAP3K11 and miR-511-3p showed that there was a significant negative correlation between them (Figure 2D). As shown in Figure 2E, the expression level of miR-511-3p in PDLSCs was increased by silencing circMAP3K11, but decreased by overexpressing circMAP3K11. These findings revealed that miR-511-3p targets circMAP3K11, suggesting the negative correlation between circMAP3K11 and miR-511-3p.

### circMAP3K11 Inhibited miR-511-3p Expression in hPDLSCs

**FIGURE 3:**
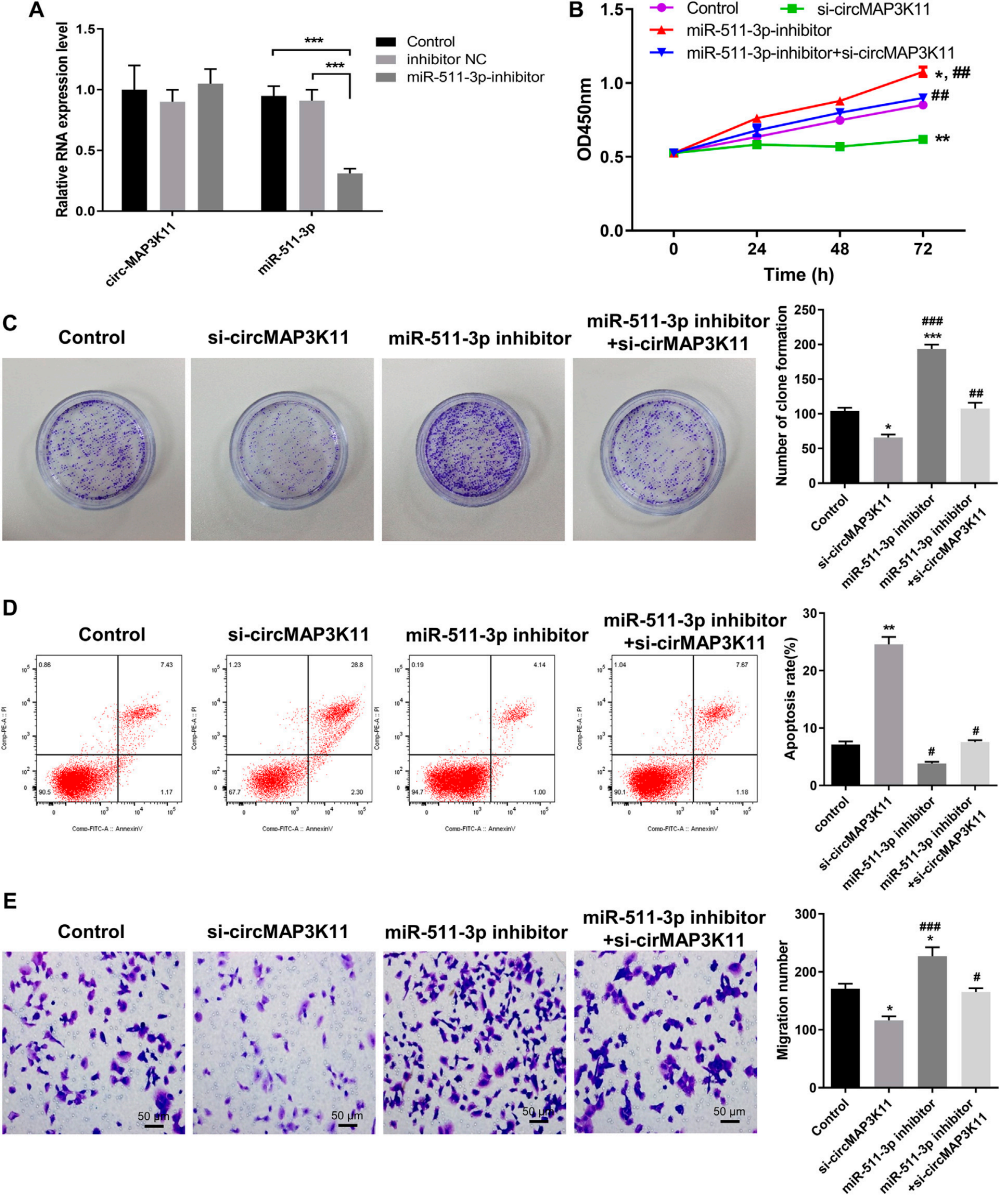
Silencing circMAP3K11 (si-circMAP3K11) antagonizes the effects of miR-511-3p inhibitor on cell viability, proliferation, apoptosis, and migration in PDLSCs **(A)** The expression of circMAP3K11 and miR-511-3p in PDLSCs transfected with miR-511-3p inhibitor and corresponding control was detected by RT-PCR **(B)** The cell viability **(C)** proliferation **(D)** cell apoptosis, and **(E)** migration ability of PDLSCs in which circMAP3K11, miR-511-3p inhibitor, or both, were repressed, were evaluated by MTT, colony formation, FCM, and transwell assays, respectively. Note: *p < 0.05, **p < 0.01, and ***p < 0.005, vs. control; #*p* < 0.05, ##*p* < 0.01, and ###*p* < 0.005, vs. miR-511-3p inhibitor.

To further investigate the regulation relationship between circMAP3K11 and miR-511-3p in hPDLSCs, hPDLSCs were treated with a miR-511-3p inhibitor. The results of RT-PCR showed that miR-511-3p was significantly downregulated compared with the inhibitor NC or control groups, while miR-511-3p inhibitor had no effect on the expression of circMAP3K11 in hPDLSCs (Figure 3A). By using MTT reagent, we detected the cell viability of hPDLSCs in which circMAP3K11, the miR-511-3p, or both, were knocked down. As shown in Figure 3B, compared to the control group, the cell viability of hPDLSCs in si-circMAP3K11 group was obviously inhibited, while that in miR-511-3p inhibitor group was obviously increased. Notably, the promotion of cell viability by miR-511-3p inhibitor was partially reversed by si-circMAP3K11. Consistently, the colony formation and Transwell assays showed that miR-511-3p inhibitor remarkably enhanced the proliferation of hPDLSCs, but this effect was blocked by si-circMAP3K11 (Figures 3C,E). In addition, the cell apoptosis of hPDLSCs was increased in miR-511-3p inhibitor group but decreased by transfection with si-circMAP3K11; moreover, the co-transfection of the miR-511-3p inhibitor and si-circMAP3K11 rescued the dysregulation (Figure 3D). These data revealed that circMAP3K11 functions to block miR-511-3p inhibition of hPDLSCs in periodontitis.

### miR-511-3p Inhibits TLR4 Expression in hPDLSCs

**FIGURE 4:**
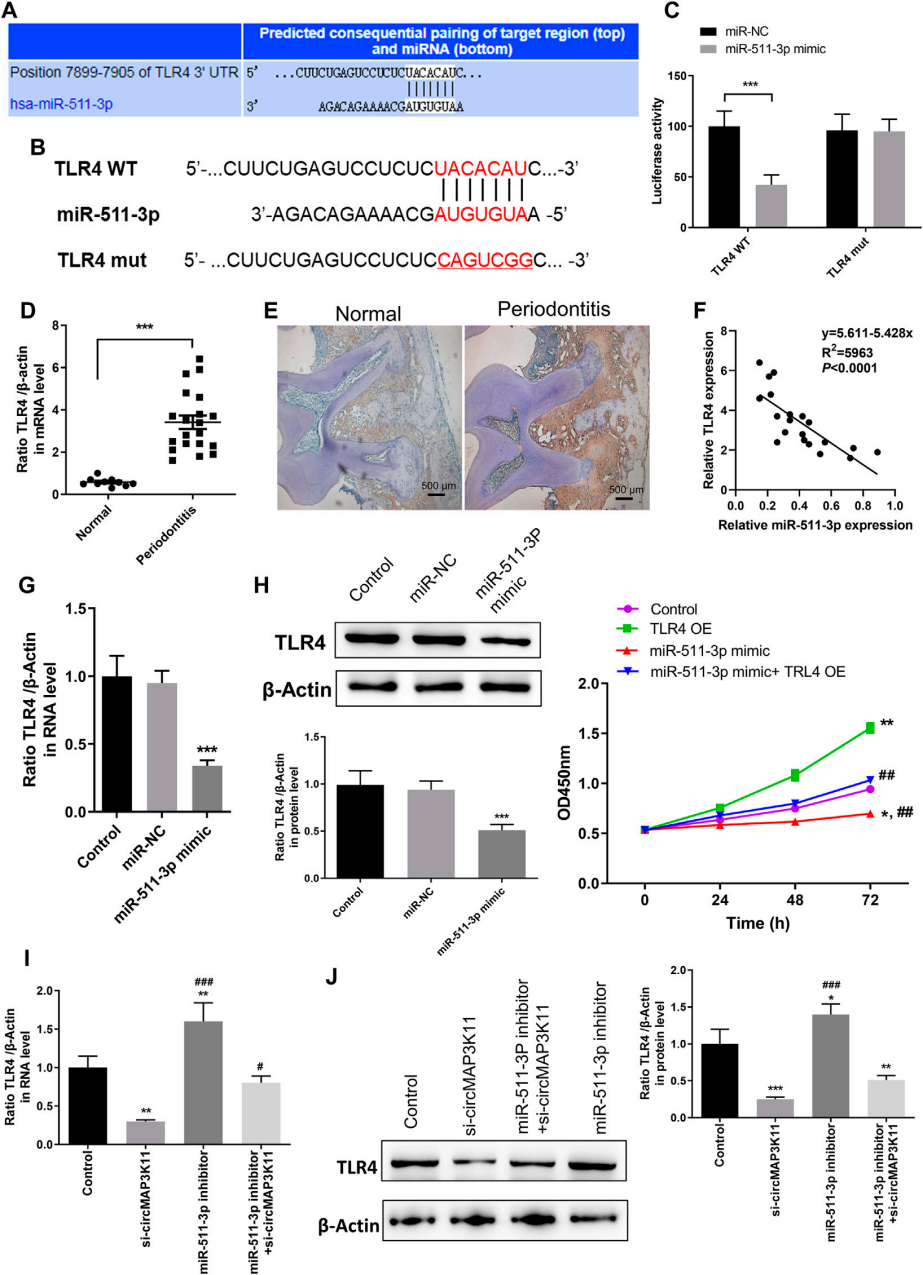
miR-511-3p is involved in periodontitis by modulating TLR4, and silencing circMAP3K11 (si-circMAP3K11) reverses the effect of miR-511-3p inhibitor on TLR4 expression in PDLSCs **(A)** The putative complementary binding sites within TLR4 and miR-511-3p predicted by TargetScan **(B)** The binding sites of miR-511-3p in the 3′UTR of TLR4, and the mutated version of the TLR4 3′UTR is also shown **(C)** The dual-luciferase reporter assay was performed to confirm that TLR4 is a direct target gene of miR-511-3p **(D)** Immunohistochemistry observed the protein expression level of TLR4 in periodontitis and normal samples **(E)** The expression level of TLR4 in periodontitis and normal samples was detected by RT-PCR **(F)** Spearman’s correlation analysis showed a negative correlation between TLR4 and miR-511-3p expression in periodontitis samples **(G)** RT-PCR examined the expression of TLR4 in PDLSCs transfected with miR-511-3p mimic or corresponding control **(H)** The promotion on cell viability in PDLSCs by TLR4 overexpression was attenuated by miR-511-3p mimic **(I)** si-circMAP3K11 reverses the effect of miR-511-3p inhibitor on TLR4 expression in PDLSCs. Note: *p < 0.05, **p < 0.01, and ***p < 0.005, vs. control; #*p* < 0.05, ##*p* < 0.01, and ###*p* < 0.005, vs. TLR4 OE or si-circMAP3K11.

The bioinformatics tool TargetScan was employed to predict the potential binding site of miR-511-3p to the 3′UTR of TLR4 (Figures 4A,B). In order to validate the interaction between miR-511-3p and TLR4, the dual-luciferase reporter assay was performed. Then, the result showed that the relative luciferase activity of cells transfected with wild type TLR4 3′UTR constructs was observably decreased by miR-511-3p mimic, whereas it was almost not changed in cells transfected with mutant TLR4 3′UTR constructs, confirming that TLR4 is a direct target of miR-511-3p (Figure 4C).

Afterward, immunohistochemistry and RT-PCR analyses were performed to investigate the expression of TLR4 in periodontal ligament tissue of periodontitis and normal patients. As shown in Figures 4D,E, TLR4 expression in periodontal ligament tissue of periodontitis group was significantly higher than that of normal group. Moreover, the correlation analysis showed that the TLR4 expression was significantly and negatively correlated with miR-511-3p expression in periodontitis (Figure 4F).

To investigate the regulation of miR-511-3p in TLR4 expression, hPDLSCs were transfected with miR-511-3p mimic. The results of RT-PCR and western blot showed that the expression of TLR4 in hPDLSCs was obviously reduced after transfection of miR-511-3p mimic (Figures 4G,H), suggesting that miR-511-3p could inhibit the expression of TLR4. Additionally, we also evaluated the viability of hPDLSCs after transfecting hPDLSCs with miR-511-3p mimic, TLR4 overexpression plasmid or their combination. As shown in Figure 4I, cell viability of hPDLSCs in control group was significantly lower than that in TLR4 overexpression group, but obviously higher than that in miR-511-3p mimic group. Besides, there was no significant difference between the control and miR-511-3p mimic + TLR4 overexpression groups in the viability of hPDLSCs, hinting that miR-511-3p mimic could limit the cell viability enhanced by TLR4 overexpression in hPDLSCs. These results signposted that TLR4 is a direct target of miR-511-3p, and the expression of TLR4 could be regulated by miR-511-3p.

### Inhibiting circMAP3K11 Reversed the Effect of miR-511-3p Inhibition on TLR4 Expression

Since the direct regulation between circMAP3K11 and miR-511-3p and that between miR-511-3p and TLR4 have been verified, we assumed that circMAP3K11 acts as miR-511-3p sponge to regulate the expression of TLR4. The results of RT-PCR and western blot showed that both the mRNA and protein expressions of TLR4 in hPDLSCs were strongly decreased after transfection of si-circMAP3K11, which was consistent with our speculation (Figures 4.J,K). Besides, miR-511-3p inhibitor transfection significantly increased the expression of TLR4 in hPDLSCs, but this enhancement was blocked by the transfection with si-circMAP3K11. These results suggested that circMAP3K11 acts as a sponge of miR-511-3p, and reverses miR-511-3p mediated TLR4 repression in hPDLSCs.

### circMAP3K11 Promoted the Proliferation and Inhibited the Apoptosis of hPDLSCs Through Functioning as miR-511-3p Sponge *in vivo*

**FIGURE 5:**
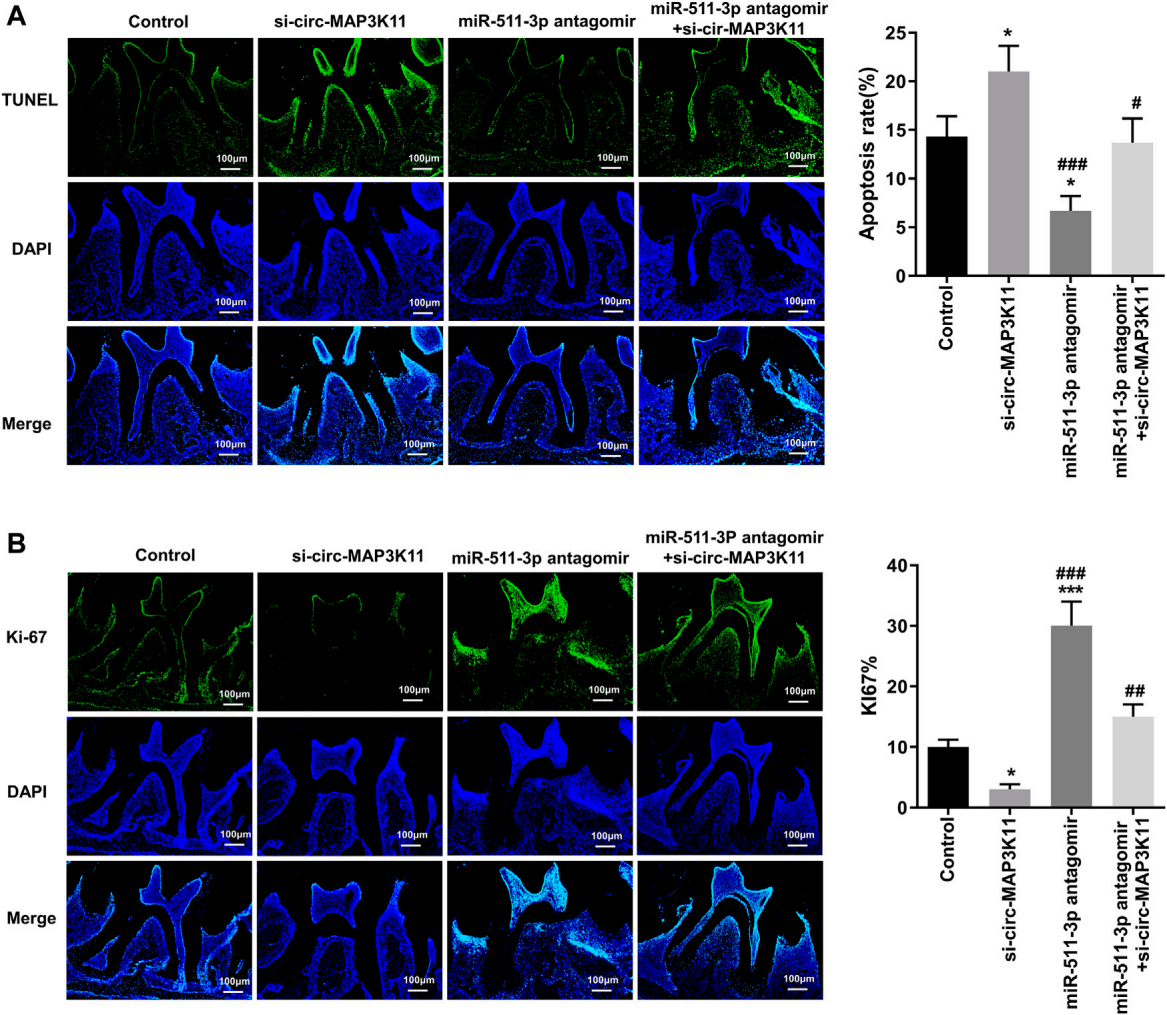
circMAP3K11 represses cell proliferation and induces cell apoptosis by sponging miR-511-3p *in vivo* **(A)** The extent of cell apoptosis and **(B)** proliferation in periodontal tissue from periodontitis mice in which circMAP3K11, miR-511-3p antagomir, or both, were repressed, were investigated respectively by using TUNEL and Ki67 assays.

To further explore the effects of circMAP3K11 and miR-511-3p on periodontitis *in vivo*, the periodontitis model was constructed by ligature-induced approach. The degree of apoptosis in periodontal tissue detected by TUNEL assay was depicted in Figure 5A. As we can see, the periodontal tissue from periodontitis mice in si-circMAP3K11 group exhibited the highest apoptosis degree among the four groups. Conversely, the rate of apoptotic cells in periodontal tissue from miR-511-3p antagomir group was significantly lower than that in periodontal tissue from other groups, and this repressive effect of miR-511-3p on apoptosis was reversed by silencing circMAP3K11. In addition, through Ki67 assay, we found that the number of proliferating cells in periodontal tissue was significantly decreased in si-circMAP3K11 group but significantly increased in miR-511-3p antagomir group when compared to the control group (Figure 5B). Notably, the effect of miR-511-3p antagomir on the proliferation of cells in periodontal tissue was hindered by the silencing of circMAP3K11. These results revealed that miR-511-3p inhibition could promote periodontitis development *in vivo*, while silencing of circMAP3K11 could reverse this result.

## Discussion

Accumulating studies on the implication of circRNAs in the development of various diseases has emerged in recent years (Lin et al., 2019; Yang et al., 2019). However, the role of circRNAs in periodontitis has not been studied so far. In the present study, we revealed that circMAP3K11 is involved in periodontitis development by regulating TLR4 expression by sponging miR-511-3p.

Initially, by using bioinformatics online tool miRanda (Enright et al., 2003) (http://www.microrna.org/microrna/home.do), we predicted circMAP3K11 as a circRNA interacting with miR-511, and its expression was elevated in periodontal ligament samples from periodontitis patients compared with those from normal subjects. PDLSCs are stem cells isolated from periodontal ligament with self-renewal ability and has multipotent capacity, which is one of the promising cell sources for periodontal regeneration (Bassir et al., 2016). A previous study demonstrated that the regenerative capacity of PDLSCs could be damaged in inflammatory microenvironment, which might be one of the pathogenesis of periodontitis (Park et al., 2011a). To explore the roles of circMAP3K11 in this process, the effects of circMAP3K11 on the PDLSCs were investigated. Functionally, silencing of circMAP3K11 could obviously hinder the viability, proliferation and migration of PDLSCs; in addition, the apoptosis of PDLSCs were induced by the silencing of circMAP3K11. On the contrary, circMAP3K11 overexpression led to inverse results. Previous studies reported that periodontitis could promote the proliferation and migration of PDLSCs (Park et al., 2011a; Zheng et al., 2015). Besides, we also detected the changes of the expression of osteoblast differentiation related proteins. The results showed that circMAP3K11 overexpression significantly reduced the protein expression of Runx2 and OSX. Runx2 is known as a master transcription factor in osteoblast and odontoblast differentiation, which is involved in the regulation of various tooth-related gene expressions (Bruderer et al., 2014). As a downstream gene of Runx2, OSX can serve as a marker for normal osteoblast differentiation (Zhou et al., 2010). Multiple studies proved that these proteins play crucial roles in periodontal regeneration (Liu et al., 2019b; Assis et al., 2020). Moreover, circMAP3K11 overexpression also repressed the expression of ATF4 and BSP. BSP is a necessary protein for cementoblast differentiation and subsequent matrix mineralization (Marom et al., 2005). ATF4 is an upstream transcriptional activator of OSX (Yu et al., 2009). These data revealed that circMAP3K11 could affect the proliferation, cell apoptosis as well as migration of PDLSCs, and might regulate OSX expression via Runx2 and ATF4 to suppress regenerative capacity of PDLSCs in periodontitis.

Afterward, we proved the binding of circMAP3K11 to miR-511-3p, as confirmed by the results of the dual luciferase reporter assay. We found a negative correlation between circMAP3K11 and miR-511-3p expression levels in tissues from periodontitis patients. The results of RT-PCR for circMAP3K11 and miR-511-3p expressions in PDLSCs with different transfections showed that the abnormal expression of circMAP3K11 could obviously affect miR-511-3p expression, whereas miR-511-3p dysregulation had no effect on circMAP3K11 expression, suggesting that miR-511-3p expression was regulated by circMAP3K11. In addition, miR-511-3p inhibitor could promote the proliferation and migration of PDLSCs, and the silencing of circMAP3K11 might reverse these effects, which confirmed the regulatory roles of circMAP3K11 on miR-511-3p expression in PDLSCs. It was previously revealed that miR-155 is expressed in periodontal tissues (Xie et al., 2011), and that the activation of macrophages is closely related to its expression (Jablonski et al., 2016). Several previous studies have proved that miR-511 has strong immune regulatory potential, and one of the most studied target genes of miR-511 is TLR4 (Tserel et al., 2011; Awuah et al., 2019). As reported, TLR4 is capable of mediating the activation of proinflammatory cytokines and worsening periodontitis (Renn et al., 2018). A previous work found that the expression of miR-511 is reduced after TLR4 stimulation with LPS (Awuah et al., 2019) and miR-511 has been shown to protect against TLR4-mediated inflammation (Awuah et al., 2020). In the current study, the dual-luciferase reporter assay confirmed that TLR4 is a direct target gene of miR-511-3p and correlation analysis, RT-PCR and western blot assays signposted that the expression of miR-511-3p is negatively correlated with TLR4 expression in periodontitis. The correlations between circMAP3K11, miR-511-3p and TLR4 were investigated based on RT-PCR and western blot results, revealing that circMAP3K11 functions as a sponge for miR-511-3p to reverse the inhibition of TLR4. Furthermore, the *in vivo* experiment further validated this finding.

Generally, circRNAs function as “miRNA sponges” to negatively regulate miRNA activity, leading to an attenuation in miRNA expression and function, which regulates the expression of the target gene (Kulcheski et al., 2016). However, this kind of molecular mechanism has seldom been studied in periodontitis before. Our study found that circMAP3K11 sponged miR-511-3p to reduce its expression, thus affecting the functions of PDLSCs. Moreover, TLR4 functions as the target protein of miR-511-3p. TLR4 has been proved to be crucial in periodontitis pathogenesis (Renn et al., 2018). A previous study indicated that TLR4 can remarkably increase inflammatory responses *in vivo*, and inhibiting TLR4 can significantly improve the symptoms of periodontitis in mice (Qi et al., 2019). Therefore, we conclude that circMAP3K11/miR-511-3p/TLR4 regulatory axis affects the functions of PDLSCs and increases the inflammatory responses in the development of periodontitis. Nevertheless, the biological function of circMAP3K11/miR-511-3p/TLR4 axis in periodontitis progression is in need of further investigation and the current study was a preliminary one to pave the way for further research in this field of science.

Collectively, our study is the first to show that the expression of TLR4 in periodontitis is regulated by a circRNA and the first to report the mechanism and clinical significance of circMAP3K11 in periodontitis. The results hinted that targeting circMAP3K11/miR-511-3p/TLR4 axis is a feasible strategy for periodontitis therapy, providing novel insights in the treatment of periodontal tissue regeneration based on stem cells.

## Data Availability

The original contributions presented in the study are included in the article/Supplementary Material, further inquiries can be directed to the corresponding author.

## References

[B1] Abbaszadeh-Goudarzi K. Radbakhsh S. Pourhanifeh M. H. Khanbabaei H. Davoodvandi A. Fathizadeh H. (2020). Circular RNA and diabetes: epigenetic regulator with diagnostic role. Curr. Mol. Med. 20 (7), 516–526. 10.2174/1566524020666200129142106. 31995005

[B2] Agarwal V. Bell G. W. Nam J. W. Bartel D. P. (2015). Predicting effective microRNA target sites in mammalian mRNAs. Elife 4, e05005. 10.7554/eLife.05005 PMC4532895 26267216

[B3] Al-Ghutaimel H. Riba H. Al-Kahtani S. Al-Duhaimi S. (2014). Common periodontal diseases of children and adolescents. Int J Dent 2014, 850674. 10.1155/2014/850674 25053946 PMC4098882

[B4] AlJehani Y. A. (2014). Risk factors of periodontal disease: review of the literature. Int J Dent 2014, 182513. 10.1155/2014/182513 24963294 PMC4055151

[B5] Assis R. F. da S. F. G. Salomão Silva M. E. Caroline da Rosário Palma I. Rovai E. S. Browne de Miranda T. (2020). Non-coding RNAs repressive role in post-transcriptional processing of RUNX2 during the acquisition of the osteogenic phenotype of periodontal ligament mesenchymal stem cells. Dev. Biol. 470, 37–48. 10.1016/j.ydbio.2020.10.012 33152274

[B6] Awuah D. Alobaid M. Latif A. Salazar F. Emes R. D. Ghaemmaghami A. M. (2019). The cross-talk between miR-511-3p and C-type lectin receptors on dendritic cells affects dendritic cell function. J. Immunol. 203 (1), 148–157. 10.4049/jimmunol.1801108 31118225

[B7] Awuah D. Ruisinger A. Alobaid M. Mbadugha C. Ghaemmaghami A. M. (2020). MicroRNA-511-3p mediated modulation of the peroxisome proliferator-activated receptor gamma (PPARγ) controls LPS-induced inflammatory responses in human monocyte derived DCs. bioRxiv 2011, 369967. 10.1101/2020.11.05.369967

[B8] Bassir S. H. Wisitrasameewong W. Raanan J. Ghaffarigarakani S. Chung J. Freire M. (2016). Potential for stem cell-based periodontal therapy. J. Cell. Physiol. 231 (1), 50–61. 10.1002/jcp.25067 26058394 PMC4627700

[B9] Bruderer M. Richards R. G. Alini M. Stoddart M. J. (2014). Role and regulation of RUNX2 in osteogenesis. Eur. Cell. Mater. 28, 269–286. 10.22203/ecm.v028a19 25340806

[B10] Enright A. J. John B. Gaul U. Tuschl T. Sander C. Marks D. S. (2003). MicroRNA targets in *Drosophila* . Genome Biol. 5 (1), R1. 10.1186/gb-2003-5-1-r1 14709173 PMC395733

[B11] Ge J. B. Lin J. T. Hong H. Y. Sun Y. J. Li Y. Zhang C. M. (2018). MiR-374b promotes osteogenic differentiation of MSCs by degrading PTEN and promoting fracture healing. Eur. Rev. Med. Pharmacol. Sci. 22 (11), 3303–3310. 10.26355/eurrev_201806_15149 29917179

[B12] Guijarro-Muñoz I. Compte M. Álvarez-Cienfuegos A. Álvarez-Vallina L. Sanz L. (2014). Lipopolysaccharide activates Toll-like receptor 4 (TLR4)-mediated NF-κB signaling pathway and proinflammatory response in human pericytes. J. Biol. Chem. 289 (4), 2457–2468. 10.1074/jbc.M113.521161 24307174 PMC3900988

[B13] Guo J. Zeng X. Miao J. Liu C. Wei F. Liu D. (2019a). MiRNA-218 regulates osteoclast differentiation and inflammation response in periodontitis rats through Mmp9. Cell Microbiol. 21 (4), e12979. 10.1111/cmi.12979 30444938

[B14] Guo P. Y. Wu L. F. Xiao Z. Y. Huang T. L. Li X. (2019b). Knockdown of MiR-140-5 promotes osteogenesis of adipose-derived mesenchymal stem cells by targeting TLR4 and BMP2 and promoting fracture healing in the atrophic nonunion rat model. Eur. Rev. Med. Pharmacol. Sci. 23 (5), 2112–2124. 10.26355/eurrev_201903_17255 30915756

[B15] Han J. Menicanin D. Gronthos S. Bartold P. M. (2014). Stem cells, tissue engineering and periodontal regeneration. Aust. Dent. J. 59 (Suppl. 1), 117–130. 10.1111/adj.12100 24111843

[B16] He X. Wang H. Jin T. Xu Y. Mei L. Yang J. (2016). TLR4 activation promotes bone marrow MSC proliferation and osteogenic differentiation via Wnt3a and Wnt5a signaling. PLoS One 11 (3), e0149876. 10.1371/journal.pone.0149876 26930594 PMC4773221

[B17] Heinsbroek S. E. Squadrito M. L. Schilderink R. Hilbers F. W. Verseijden C. Hofmann M. (2016). miR-511-3p, embedded in the macrophage mannose receptor gene, contributes to intestinal inflammation. Mucosal Immunol. 9 (4), 960–973. 10.1038/mi.2015.113 26530135

[B18] Huang C. Y. Pelaez D. Dominguez-Bendala J. Bendala J. D. Garcia-Godoy F. Cheung H. S. (2009). Plasticity of stem cells derived from adult periodontal ligament. Regen. Med. 4 (6), 809–821. 10.2217/rme.09.55 19903001

[B19] Hynes K. Menicanin D. Gronthos S. Bartold P. M. (2012). Clinical utility of stem cells for periodontal regeneration. Periodontol. 2000 59 (1), 203–227. 10.1111/j.1600-0757.2012.00443.x 22507067

[B20] Jablonski K. A. Gaudet A. D. Amici S. A. Popovich P. G. Guerau-de-Arellano M. (2016). Control of the inflammatory macrophage transcriptional signature by miR-155. PLoS One 11 (7), e0159724. 10.1371/journal.pone.0159724 27447824 PMC4957803

[B21] Janssens S. Beyaert R. (2003). Role of Toll-like receptors in pathogen recognition. Clin. Microbiol. Rev. 16 (4), 637–646. 10.1128/cmr.16.4.637-646.2003 14557290 PMC207104

[B22] Kebschull M. Papapanou P. N. (2015). Mini but mighty: microRNAs in the pathobiology of periodontal disease. Periodontol. 2000 69 (1), 201–220. 10.1111/prd.12095 26252410 PMC4530521

[B23] Kinane D. F. Stathopoulou P. G. Papapanou P. N. (2017). Periodontal diseases. Nat Rev Dis Primers 3, 17038. 10.1038/nrdp.2017.38 28805207

[B24] Kizil C. Kyritsis N. Brand M. (2015). Effects of inflammation on stem cells: together they strive?. EMBO Rep. 16 (4), 416–426. 10.15252/embr.201439702 25739812 PMC4388609

[B25] Koning J. J. Kooij G. de Vries H. E. Nolte M. A. Mebius R. E. (2013). Mesenchymal stem cells are mobilized from the bone marrow during inflammation. Front. Immunol. 4, 49. 10.3389/fimmu.2013.00049 23459632 PMC3586765

[B26] Kulcheski F. R. Christoff A. P. Margis R. (2016). Circular RNAs are miRNA sponges and can be used as a new class of biomarker. J. Biotechnol. 238, 42–51. 10.1016/j.jbiotec.2016.09.011 27671698

[B27] Kulkarni V. Bhatavadekar N. B. Uttamani J. R. (2014). The effect of nutrition on periodontal disease: a systematic review. J. Calif. Dent. Assoc. 42 (5), 302–311 25087348

[B28] Lee Y. H. Na H. S. Jeong S. Y. Jeong S. H. Park H. R. Chung J. (2011). Comparison of inflammatory microRNA expression in healthy and periodontitis tissues. Biocell 35 (2), 43–49. 10.32604/BIOCELL.2011.35.043 22128589

[B29] Li C. Li B. Dong Z. Gao L. He X. Liao L. (2014). Lipopolysaccharide differentially affects the osteogenic differentiation of periodontal ligament stem cells and bone marrow mesenchymal stem cells through Toll-like receptor 4 mediated nuclear factor κB pathway. Stem Cell Res. Ther. 5 (3), 67. 10.1186/scrt456 24887697 PMC4076620

[B30] Lin F. Zhao G. Chen Z. Wang X. Lv F. Zhang Y. (2019). circRNA-miRNA association for coronary heart disease. Mol. Med. Rep. 19 (4), 2527–2536. 10.3892/mmr.2019.9905 30720076 PMC6423602

[B31] Liu J. Wang L. Liu W. Li Q. Jin Z. Jin Y. (2014). Dental follicle cells rescue the regenerative capacity of periodontal ligament stem cells in an inflammatory microenvironment. PloS One 9 (9), e108752. 10.1371/journal.pone.0108752 25275580 PMC4183515

[B32] Liu W. Zheng Y. Chen B. Ke T. Shi Z. (2019a). LncRNA papillary thyroid carcinoma susceptibility candidate 3 (PTCSC3) regulates the proliferation of human periodontal ligament stem cells and toll-like receptor 4 (TLR4) expression to improve periodontitis. BMC Oral Health 19 (1), 108. 10.1186/s12903-019-0802-9 31196168 PMC6567910

[B33] Liu X. Niu Y. Xie W. Wei D. Du Q. (2019b). Tanshinone IIA promotes osteogenic differentiation of human periodontal ligament stem cells via ERK1/2-dependent Runx2 induction. Am J Transl. Res. 11 (1), 340–350 30787991 PMC6357334

[B34] Liu Y. Zheng Y. Ding G. Fang D. Zhang C. Bartold P. M. (2008). Periodontal ligament stem cell-mediated treatment for periodontitis in miniature swine. Stem Cell. 26 (4), 1065–1073. 10.1634/stemcells.2007-0734 PMC2653213 18238856

[B35] Liu Z. He Y. Xu C. Li J. Zeng S. Yang X. (2020). The role of PHF8 and TLR4 in osteogenic differentiation of periodontal ligament cells in inflammatory environment. J. Periodontol. 10.1002/jper.20-0285 33040333

[B36] Livak K. J. Schmittgen T. D. (2001). Analysis of relative gene expression data using real-time quantitative PCR and the 2(-Delta Delta C(T)) method. Methods 25 (4), 402–408. 10.1006/meth.2001.1262 11846609

[B37] Luan X. Zhou X. Trombetta-eSilva J. Francis M. Gaharwar A. K. Atsawasuwan P. (2017). MicroRNAs and periodontal homeostasis. J. Dent. Res. 96 (5), 491–500. 10.1177/0022034516685711 28068481 PMC5453493

[B38] Marom R. Shur I. Solomon R. Benayahu D. (2005). Characterization of adhesion and differentiation markers of osteogenic marrow stromal cells. J. Cell. Physiol. 202 (1), 41–48. 10.1002/jcp.20109 15389528

[B39] Mourkioti F. Rosenthal N. (2005). IGF-1, inflammation and stem cells: interactions during muscle regeneration. Trends Immunol. 26 (10), 535–542. 10.1016/j.it.2005.08.002 16109502

[B40] Park J. C. Kim J. M. Jung I. H. Kim J. C. Choi S. H. Cho K. S. (2011a). Isolation and characterization of human periodontal ligament (PDL) stem cells (PDLSCs) from the inflamed PDL tissue: *in vitro* and *in vivo* evaluations. J. Clin. Periodontol. 38 (8), 721–731. 10.1111/j.1600-051X.2011.01716.x 21449989

[B41] Park J. Y. Jeon S. H. Choung P. H. (2011b). Efficacy of periodontal stem cell transplantation in the treatment of advanced periodontitis. Cell Transplant. 20 (2), 271–285. 10.3727/096368910x519292 20719084

[B42] Pekovic V. Hutchison C. J. (2008). Adult stem cell maintenance and tissue regeneration in the ageing context: the role for A-type lamins as intrinsic modulators of ageing in adult stem cells and their niches. J. Anat. 213 (1), 5–25. 10.1111/j.1469-7580.2008.00928.x 18638067 PMC2475560

[B43] Potnis P. A. Dutta D. K. Wood S. C. (2013). Toll-like receptor 4 signaling pathway mediates proinflammatory immune response to cobalt-alloy particles. Cell. Immunol. 282 (1), 53–65. 10.1016/j.cellimm.2013.04.003 23680697

[B44] Qi W. Yang X. Ye N. Li S. Han Q. Huang J. (2019). TLR4 gene in the regulation of periodontitis and its molecular mechanism. Exp. Ther. Med. 18 (3), 1961–1966. 10.3892/etm.2019.7809 31452696 PMC6704533

[B45] Renn T. Y. Huang Y. K. Feng S. W. Wang H. W. Lee W. F. Lin C. T. (2018). Prophylactic supplement with melatonin successfully suppresses the pathogenesis of periodontitis through normalizing RANKL/OPG ratio and depressing the TLR4/MyD88 signaling pathway. J. Pineal Res. 64 (3), e12464. 10.1111/jpi.12464 29274168

[B46] Seo B. M. Miura M. Gronthos S. Bartold P. M. Batouli S. Brahim J. (2004). Investigation of multipotent postnatal stem cells from human periodontal ligament. Lancet 364 (9429), 149–155. 10.1016/s0140-6736(04)16627-0 15246727

[B47] Sun Y. Shu R. Zhang M. Z. Wu A. P. (2008). Toll-like receptor 4 signaling plays a role in triggering periodontal infection. FEMS Immunol. Med. Microbiol. 52 (3), 362–369. 10.1111/j.1574-695X.2008.00386.x 18328075

[B48] Tserel L. Runnel T. Kisand K. Pihlap M. Bakhoff L. Kolde R. (2011). MicroRNA expression profiles of human blood monocyte-derived dendritic cells and macrophages reveal miR-511 as putative positive regulator of Toll-like receptor 4. J. Biol. Chem. 286 (30), 26487–26495. 10.1074/jbc.M110.213561 21646346 PMC3143613

[B49] VanHook A. M. (2019). Inflammation induces stem cell quiescence. Sci. Signal. 12 (605), eaaz9665. 10.1126/scisignal.aaz9665

[B50] Wei F. Yang S. Guo Q. Zhang X. Ren D. Lv T. (2017). MicroRNA-21 regulates osteogenic differentiation of periodontal ligament stem cells by targeting Smad5. Sci. Rep. 7 (1), 16608. 10.1038/s41598-017-16720-8 29192241 PMC5709498

[B51] Xie Y. F. Shu R. Jiang S. Y. Liu D. L. Zhang X. L. (2011). Comparison of microRNA profiles of human periodontal diseased and healthy gingival tissues. Int. J. Oral Sci. 3 (3), 125–134. 10.4248/ijos11046 21789961 PMC3470093

[B52] Yang H. Gao L. N. An Y. Hu C. H. Jin F. Zhou J. (2013). Comparison of mesenchymal stem cells derived from gingival tissue and periodontal ligament in different incubation conditions. Biomaterials 34 (29), 7033–7047. 10.1016/j.biomaterials.2013.05.025 23768902

[B53] Yang X. Liu L. Zou H. Zheng Y. W. Wang K. P. (2019). circZFR promotes cell proliferation and migration by regulating miR-511/AKT1 axis in hepatocellular carcinoma. Dig. Liver Dis. 51 (10), 1446–1455. 10.1016/j.dld.2019.04.012 31147216

[B54] Yoshihara-Hirata C. Yamashiro K. Yamamoto T. Aoyagi H. Ideguchi H. Kawamura M. (2018). Anti-HMGB1 neutralizing antibody attenuates periodontal inflammation and bone resorption in a murine periodontitis model. Infect. Immun. 86 (5), e00111–e00118. 10.1128/IAI.00111-18 29531138 PMC5913859

[B55] Yu B. Wang Z. (2014). Effect of concentrated growth factors on beagle periodontal ligament stem cells *in vitro* . Mol. Med. Rep. 9 (1), 235–242. 10.3892/mmr.2013.1756 24173502

[B56] Yu S. Franceschi R. T. Luo M. Fan J. Jiang D. Cao H. (2009). Critical role of activating transcription factor 4 in the anabolic actions of parathyroid hormone in bone. PloS One 4 (10), e7583. 10.1371/journal.pone.0007583 19851510 PMC2762317

[B57] Zhang H. D. Jiang L. H. Sun D. W. Hou J. C. Ji Z. L. (2018). CircRNA: a novel type of biomarker for cancer. Breast Cancer 25 (1), 1–7. 10.1007/s12282-017-0793-9 28721656

[B58] Zheng J. Zhu X. He Y. Hou S. Liu T. Zhi K. (2021). CircCDK8 regulates osteogenic differentiation and apoptosis of PDLSCs by inducing ER stress/autophagy during hypoxia. Ann. N. Y. Acad. Sci. 1485 (1), 56–70. 10.1111/nyas.14483 32978798

[B59] Zheng W. Wang S. Wang J. Jin F. (2015). Periodontitis promotes the proliferation and suppresses the differentiation potential of human periodontal ligament stem cells. Int. J. Mol. Med. 36 (4), 915–922. 10.3892/ijmm.2015.2314 26310866 PMC4564090

[B60] Zhou X. Zhang Z. Feng J. Q. Dusevich V. M. Sinha K. Zhang H. (2010). Multiple functions of Osterix are required for bone growth and homeostasis in postnatal mice. Proc. Natl. Acad. Sci. U.S.A. 107 (29), 12919–12924. 10.1073/pnas.0912855107 20615976 PMC2919908

